# Inhibition of PTP1B disrupts cell–cell adhesion and induces anoikis in breast epithelial cells

**DOI:** 10.1038/cddis.2017.177

**Published:** 2017-05-11

**Authors:** Bylgja Hilmarsdottir, Eirikur Briem, Skarphedinn Halldorsson, Jennifer Kricker, Sævar Ingthorsson, Sigrun Gustafsdottir, Gunhild M Mælandsmo, Magnus K Magnusson, Thorarinn Gudjonsson

**Affiliations:** 1 Stem Cell Research Unit, Department of Medical Faculty, Biomedical Center, School of Health Sciences, University of Iceland, Reykjavik, Iceland; 2 Department of Tumor Biology, Institute for Cancer Research, The Norwegian Radium Hospital, Oslo University Hospital Nydalen, Oslo, Norway; 3 Department of Laboratory Hematology Landspitali, University Hospital, Reykjavik, Iceland; 4 System Biology Center, University of Iceland, Reykjavik, Iceland

## Abstract

Protein tyrosine phosphatase 1B (PTP1B) is a well-known inhibitor of insulin signaling pathways and inhibitors against PTP1B are being developed as promising drug candidates for treatment of obesity. PTP1B has also been linked to breast cancer both as a tumor suppressor and as an oncogene. Furthermore, PTP1B has been shown to be a regulator of cell adhesion and migration in normal and cancer cells. In this study, we analyzed the PTP1B expression in normal breast tissue, primary breast cells and the breast epithelial cell line D492. In normal breast tissue and primary breast cells, PTP1B is widely expressed in both epithelial and stromal cells, with highest expression in myoepithelial cells and fibroblasts. PTP1B is widely expressed in branching structures generated by D492 when cultured in 3D reconstituted basement membrane (3D rBM). Inhibition of PTP1B in D492 and another mammary epithelial cell line HMLE resulted in reduced cell proliferation and induction of anoikis. These changes were seen when cells were cultured both in monolayer and in 3D rBM. PTP1B inhibition affected cell attachment, expression of cell adhesion proteins and actin polymerization. Moreover, epithelial to mesenchymal transition (EMT) sensitized cells to PTP1B inhibition. A mesenchymal sublines of D492 and HMLE (D492M and HMLEmes) were more sensitive to PTP1B inhibition than D492 and HMLE. Reversion of D492M to an epithelial state using miR-200c-141 restored resistance to detachment induced by PTP1B inhibition. In conclusion, we have shown that PTP1B is widely expressed in the human breast gland with highest expression in myoepithelial cells and fibroblasts. Inhibition of PTP1B in D492 and HMLE affects cell–cell adhesion and induces anoikis-like effects. Finally, cells with an EMT phenotype are more sensitive to PTP1B inhibitors making PTP1B a potential candidate for further studies as a target for drug development in cancer involving the EMT phenotype.

Protein tyrosine phosphatases (PTPs) and tyrosine kinases modulate cellular levels of tyrosine phosphorylation and regulate many cellular events such as differentiation, cell growth, motility and proliferation.^1^ Regulation of the balance between tyrosine phosphorylation and dephosphorylation within cells is important for many cellular processes and homeostasis and is implicated in a number of human diseases.^2^

Protein tyrosine phosphatase 1B (PTP1B) is a 50 kDa non-receptor phosphatase localized predominantly on the cytoplasmic surface of the endoplasmic reticulum, anchored via its C-terminal region.^3^ PTP1B has a major role in downregulating insulin and leptin signaling^4^ by dephosphorylating the insulin receptor and thus terminating its signals. PTP1B-deficient mice are hypersensitive to insulin and resistant to obesity induced by a calorie-rich diet.^5^ For this reason, PTP1B has received attention over the last few years as a novel therapeutic target for the treatment of diabetes and obesity, and as such there are numerous inhibitors against PTP1B at various stages of development.^6^

In addition to insulin regulation, PTP1B also has a role in other signaling pathways, such as growth factor and integrin mediated processes, as well as cancer development.^7, 8^ PTP1B is a major activator of Src by dephosphorylating the inhibitory tyrosine phosphorylation site (Y529) on the COOH terminus of the kinase.^9^ PTP1B has been shown to be a positive mediator of the ErbB2-induced signals that trigger breast tumorigenesis^10, 11^ and to be required for ErbB2 transformation in breast epithelial cells through Src activation.^12^ Substrate trapping and biochemical studies have identified various substrates of PTP1B involved in cell adherence and matrix attachment. For example, PTP1B regulates the intracellular protein tyrosine kinases like focal adhesion kinase (FAK), Src and adaptor proteins like *β*-catenin and p130Cas.^13, 14, 15^ PTP1B has also been shown to interact with integrin complexes and localize to early cell matrix adhesion sites.^3^ Mouse fibroblasts, which expressed a dominant-negative form of PTP1B have small matrix sites, are impaired in cell spreading and show reduced Src activity. Similarly, cells derived from PTP1B KO mice also show defects in cell spreading.^16^ These data suggest that PTP1B is an important regulator of integrin signaling pathways thereby indicating a role in adhesion, spreading and formation of focal adhesion.^17^ Developmental events underlying branching morphogenesis in the breast are closely related to pathways important for cancer progression, that is, epithelial plasticity and epithelial–mesenchymal transition (EMT). EMT is a process where epithelial cells lose epithelial characteristics and gain mesenchymal traits. Although still debated, EMT is widely considered to be a key process in metastatic breast cancer, which is the leading cause of cancer deaths.^18^ During EMT, intracellular contacts change and during the transformation from a polarized epithelium to the mesenchymal state there is loss of tight junctions and adhesion molecules.^19^ EMT is believed to have an important role in cancer progression, invasion, formation of metastasis and resistance to cancer treatment.^20^ Thus, identification of drugs that specifically target cells with the EMT phenotype, could be a key event to improve cancer treatment.

In light of its central role in cell adhesion and Src regulation, as well as its potential role in cancer, we sought to investigate the impact of PTP1B inhibition on breast epithelial and mesenchymal cells. Inhibiting PTP1B in D492 and HMLE, breast epithelial cell lines, leads to apoptotic cell death with morphological characteristics of anoikis, cell death induced by loss of extracellular matrix attachment. PTP1B inhibition leads to downregulation of cell adhesion proteins and disrupted actin polymerization. In addition, we show that the transition from an epithelial to a mesenchymal state sensitizes mammary cells to PTP1B inhibition, suggesting the therapeutic potential of PTP1B inhibition in cancer treatment.

## Results

### PTP1B is ubiquitously expressed in normal human breast tissue

**Figure 1:**
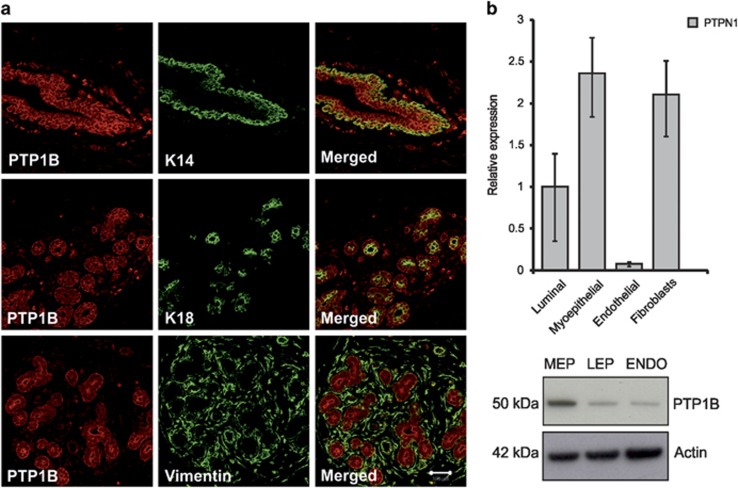
PTP1B is highly expressed in normal human breast tissue. (**a**) PTP1B is expressed both in the epithelial and stromal compartments of the breast. Immunohistochemical stainings show prominent co-expression of PTP1B (red) and K14 (green) in myoepithelial cells (top row), PTP1B (red) and K18 (green) in luminal epithelial cells (middle row), PTP1B (red) and vimentin (green) stains myoepithelial and fibroblasts (bottom row). Bar 100 *μ*m. (**b**) PTP1B is predominantly expressed in myoepithelial cells and fibroblasts in the breast gland. qPCR mRNA analysis shows that PTP1B expression is higher in myoepithelial cells (MEP) and fibroblasts than in luminal epithelial cells (LEP) and endothelial cells (ENDO). Expression levels were normalized to GAPDH (upper). Western blot confirmed high expression of PTP1B in myoepithelial cells (lower). Actin was used as a loading control

PTP1B has in recent years been linked to both tumorigenesis and tumor suppression^21, 22^ but less is known about its expression and function in the normal human breast gland. Histochemical analysis of paraffin-embedded breast tissue with antibodies against PTP1B and cell type-specific markers, K14 (myoepithelial), K18 (luminal epithelial) and vimentin (myoepithelial and fibroblasts), revealed that PTP1B is, at least partially, co-stained with each of these markers (Figure 1a). The expression intensity, however, varied between different cell populations. Isolated cell populations derived from reduction mammoplasty show that PTP1B expression is enriched in myoepithelial cells and fibroblasts (Figure 1b). Thus, PTP1B is ubiquitously expressed in epithelial cells in the breast, as well as in stroma.

### PTP1B inhibition results in apoptosis of breast epithelial cells

**Figure 2:**
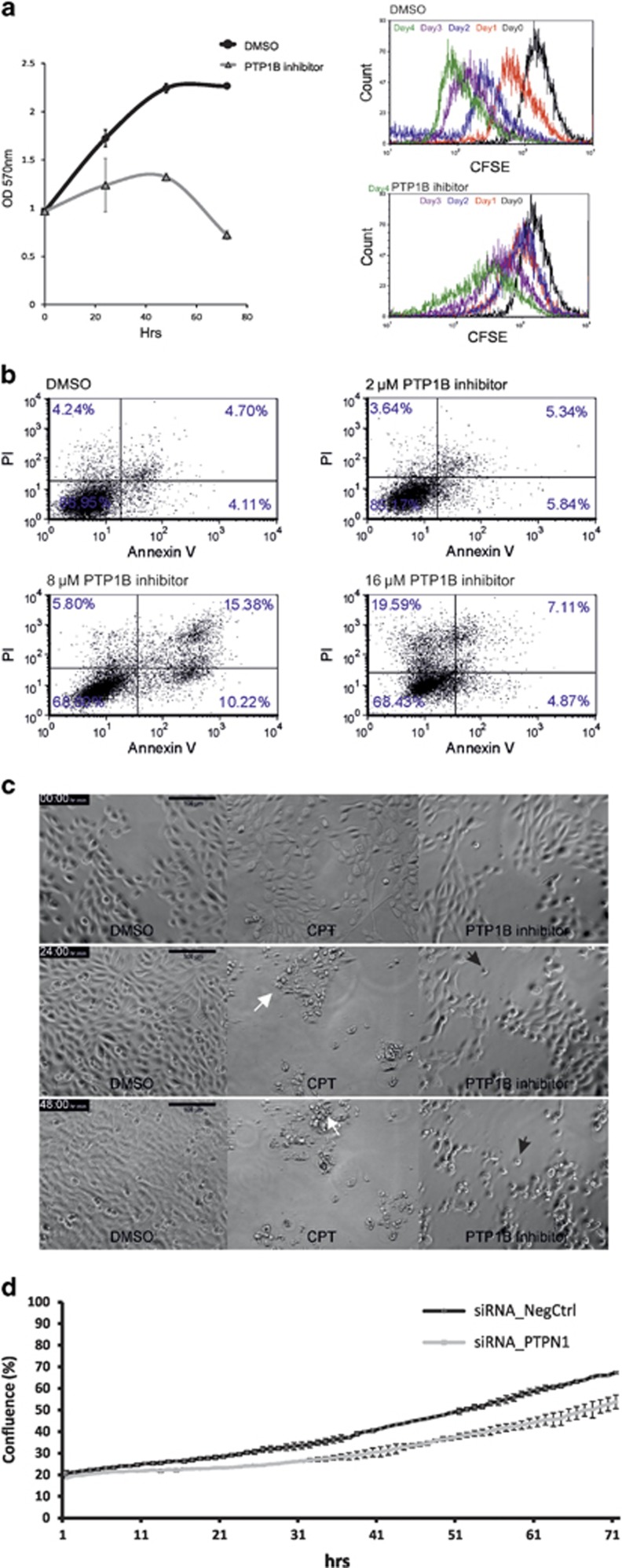
PTP1B is important for proliferation and survival of breast epithelial cells. (**a**) Inhibition of PTP1B reduces cell division and affects survival of D492 cells. Left cell survival assay reveals that PTP1B inhibitor causes reduction in proliferation and cell death in D492. D492 were treated with DMSO (control) or 16 *μ*M of a specific PTP1B inhibitor for 3 days, stained with crystal violet and the optical density at 570 nm determined. All experiments were conducted in triplicate. Right, D492 cells were stained with CFSE fluorescent dye, treated with DMSO or 8 *μ*M of PTP1B inhibitor and analyzed using flow cytometry over a period of 4 days. Note, reduced cell division in D492 after PTP1B inhibition. (**b**) Inhibition of PTP1B results in apoptosis of D492 cells. Annexin V and PI staining shows that PTP1B inhibitor induces apoptotic cell death in D492. D492 cells were treated with DMSO (control) and various concentrations of PTP1B inhibitor. (**c**) Cell death induced by the PTP1B inhibitor shows the characteristics of anoikis. CPT induces classical apoptosis of D492 cells over time in culture (middle column). D492 cells were treated with DMSO as a control (left column). Treatment with PTP1B inhibitor results in failure of cells to establish a confluent monolayer with a number of cells forming a rounded detached appearance as in cells undergoing anoikis. (**d**) Knockdown of PTPN1 reduces proliferation of D492 cells. D492 cells were transfected with siRNA PTPN1 s11507 or negative control no. 1 siRNA at 50 nM and cell confluence was monitored for 72 h using IncuCyte Zoom Live Cell Analysis System. Knockdown of PTPN1 resulted in an inhibition of confluency, whereby D492 cells treated with siRNA against PTPN1 demonstrated significantly less cell proliferation than cells treated with negative control siRNA. All experiments were done in triplicate

**Figure 3:**
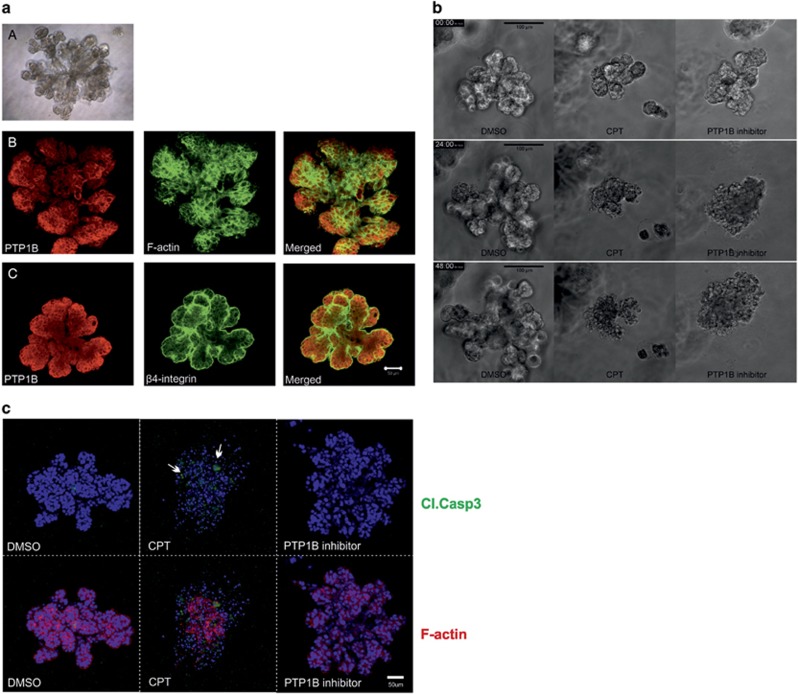
Inhibition of PTP1B affects branching morphogenesis and induces anoikis-like effects in D492 cells cultured in 3D. (**a**) Expression of PTP1B in TDLU-like structures generated by D492 cells in 3D culture. (A) D492 cells form branching structures when cultured in 3D rBM matrix. (B) Expression of F-actin (green), PTP1B (red) and (C) expression of *β*4-integrin (green) and PTP1B in D492 cells cultured in 3D rBM. Note, the strong expression of PTP1B at the lobular ends. Bar 50 *μ*m. (**b**) Inhibition of PTP1B induces loss of cell adhesion in 3D structures. D492 cells cultured in 3D rBM were treated with 10 *μ*M of CPT, 32 *μ*M of the PTP1B inhibitor or DMSO as control for 48 h. (**c**) D492 cells treated with PTP1B inhibitor die by anoikis. D492 cells treated with the PTP1B inhibitor do not show staining for cleaved caspase 3 in 3D culture (green). CPT was used as a positive control for apoptosis, arrows point to cells positive for cleaved caspase 3. Phalloidin stains F-actin (red). DAPI was used for nuclear staining (blue)

Given the ubiquitous expression of PTP1B in the human breast gland, we examined if PTP1B had a role in proliferation and survival of breast epithelial cells. D492 is a breast epithelial cell line with stem cell properties that is able to generate luminal and myoepithelial cells and generates branching structures reminiscent of terminal duct lobular units (TDLUs) when cultured in 3D reconstituted basement membrane (3D rBM; Figure 3a). To test the effects of the inhibition of PTP1B in D492, a specific PTP1B inhibitor (IC_50_=8*μ*M) against the catalytic domain of the protein was used. When cell growth and proliferation were analyzed, PTP1B inhibition had a major effect on the survival of D492. After 72 h of treatment with 16 *μ*M of the PTP1B inhibitor, the majority of D492 cells were dead (Figure 2a, left). To estimate the effect of PTP1B inhibition on the proliferation rate of D492 cells, we performed a cell tracing experiment using carboxyfluorescein succinimidyl ester (CFSE). CFSE is a fluorescent membrane dye that measures cell proliferation by flow cytometry. D492 cells were treated with either a DMSO control or 8 *μ*M of the PTP1B inhibitor. Flow cytometry analysis of the CFSE-stained cells showed that 8 *μ*M of PTP1B inhibitor substantially reduced cell division of D492 cells opposed to the DMSO-treated cells (Figure 2a, right). To further validate our findings, we tested the effect of PTP1B inhibition on two additional cell lines, MCF10a and HMLE (Supplementary Figures 1A and B). Treatment of both of these cell lines with the PTP1B inhibitor resulted in severe reduction of cellular growth and cell death, similar to what was observed in D492 cells.

Annexin V apoptosis assay demonstrates that PTP1B inhibition induces apoptosis in D492 (Figure 2b). In cells treated with DMSO or a low concentration of the PTP1B inhibitor (2 *μ*M), only low levels of annexin V and propidium iodide (PI) were detected. In contrast, when D492 cells were treated with 8 *μ*M of the PTP1B inhibitor, 26% of the cells were annexin V positive and 21% PI positive, of which 15% were stained for both markers. Annexin V/PI staining of cells with increasing PTP1B inhibitor concentration demonstrates several stages of programmed cell death, from early to late apoptosis. The highest level of cell death was observed when D492 cells were treated with 16 *μ*M of the PTP1B inhibitor. Annexin V/PI staining indicates that the cells are in late apoptosis where 27% of cells stain positive for PI and only 12% are positive for annexin V. Interestingly, these apoptosis-inducing effects were not seen if experiments were done in medium containing serum or serum supplements (Supplementary Figure 3) indicating that this inhibitor is not effective in serum-containing media. To supplement our work with the inhibitor, we also knocked down PTP1B using siRNA (Figure 2d). This resulted in an inhibition of confluency, whereby D492 cells treated with siRNA against PTP1B demonstrated significantly less cell proliferation than cells treated with negative control siRNA. Moreover, addition of serum did not rescue cell survival after PTP1B inhibition using siRNA (Supplementary Figure 3b). These results indicate that D492 cells are induced to undergo programmed cell death after inhibition of PTP1B, whether it is via a small molecule inhibitor or siRNA knockdown of PTP1B.

The classical morphology of a cell going through apoptosis is nuclear fragmentation, cell shrinkage and membrane blebbing.^23^ The observed morphology of cells after treatment with the PTP1B inhibitor was not consistent with these hallmarks of apoptosis. To compare the morphology of cells undergoing classic apoptosis and cell death induced by PTP1B inhibition, D492 cells were treated with 10 *μ*M camptothecin (CPT), an apoptosis inducer alongside the PTP1B inhibitor. Cells treated with CPT show typical apoptosis morphology (Figure 2c and Supplementary Video File 1), whereas cells treated with the PTP1B inhibitor appear round and surrounded by a halo as they detach from the surface. This morphology is consistent with cells undergoing anoikis, programmed cell death induced by detachment from the extracellular matrix.^24, 25, 26^

### Inhibition of PTP1B in D492 cells results in disturbed morphogenesis suggesting anoikis

D492 cells generate *in vivo*-like branching structures in 3D cell culture (Figure 3a, top). Analysis of PTP1B expression in these branching structures revealed similar widespread expression as in the normal breast gland with increased intensity at the lobular ends (Figure 3a). F-actin is a cytoskeletal protein that is expressed equally throughout the structure, whereas *β*4-integrin is localized at the surface of the structure in contact with the basement membrane.

D492 branching structures were treated with the 32 *μ*M of the PTP1B inhibitor, or 20 *μ*M of CPT as control for 48 h. Figure 3b (see also Supplementary Video File 2) shows that while CPT induces cell blebbing, shrinkage and accumulation of cell debris surrounding the structure, the PTP1B inhibitor induces loss of cell–cell contact and the branching structure shows a ‘grape-like’ morphology where cells do not move synchronically within the structure (as in the control), but rather individually.

In-gel immunostaining of treated branching structures shows that only CPT treatment induces cleaved caspase-3 staining (Figure 3c). Staining for of F-actin is diminished in structures treated with PTP1B inhibitor compared with DMSO treatment.

### Src activity in D492 cells is dependent on PTP1B

**Figure 4:**
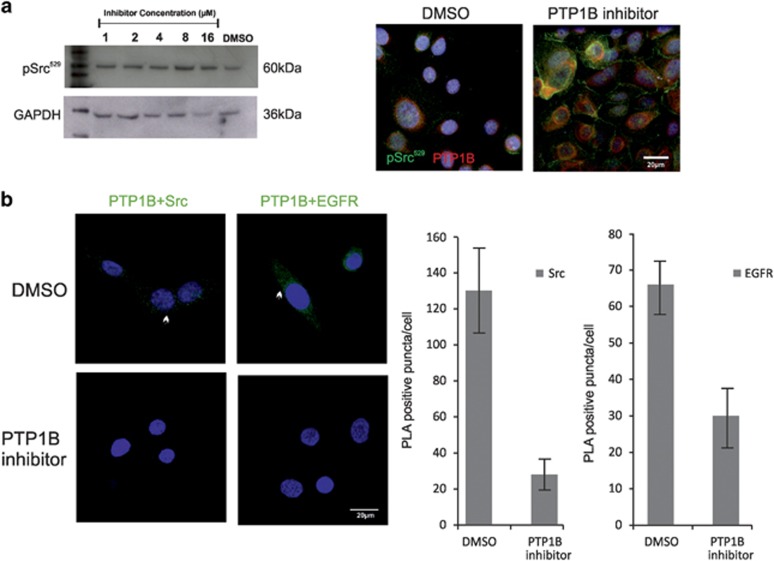
PTP1B inhibitor decreases Src activation in D492 cells. (**a**) Treatment with PTP1B inhibitor results in increased phosphorylation of the inhibitory SrcY^529^. Left, western blot analysis of pSrc^Y529^ shows that PTP1B inhibitor increases phosphorylation of pSrcY^529^. Protein extracts from D492 cells after treatment with DMSO and various concentrations of PTP1B inhibitor for 48 h. GAPDH as loading control. Right, Immunofluorescence staining of pSrc in D492 cells treated with the PTP1B inhibitor confirms increased phosphorylation of pSrc^Y529^ after PTP1B inhibition. pSrc (green), PTP1B (red), TOPRO-3 nuclear staining (blue). Bar 20 *μ*m. (**b**) Inhibition of PTP1B disrupts PTP1B protein interactions. PLA shows that PTP1B complexes with Src (left) and EGFR (right) (green arrows) are disassembled (left). DAPI nuclear stain (blue). Cell profiler was used to quantify frequency of positive puncta per cell (right)

Src activity is regulated by phosphorylation of two distinct tyrosine residues, autophosphorylation of Tyr^416^ in the kinase domain activates Src whereas phosphorylation of Tyr^529^ in the C-terminal tail inactivates it. Consequently, activation of Src requires the removal of the C-terminal phosphate by specific PTPs. To assess the effect of PTP1B inhibition on Src phosphorylation in D492 cells, we treated the cells with various concentrations of the PTP1B inhibitor for 48 h followed by protein isolation for western blot analysis. The phosphorylation level at Tyr^529^ was determined using a phosphospecific antibody as shown in Figure 4. Treatment of D492 cells with the PTP1B inhibitor caused a significant increase in Tyr^529^ phosphorylation (Figure 4a). Phosphorylation on the inhibitory tyrosine increased gradually with increased concentration of the inhibitor. To support this finding, D492 cells were treated with DMSO or 16 *μ*M of the PTP1B inhibitor for 3 h. The cells were then fixed with methanol and stained for PTP1B and pSrc Tyr^529^. The phosphorylation status of Src had increased drastically and appeared to reside at the cell membrane (Figure 4a, right).

To confirm that the PTP1B inhibitor is working directly on PTP1B interactions with substrate proteins, we used proximity ligation assay (PLA), which detects localization of protein interactions within the cell. Using antibodies detecting EGFR and Src (known PTP1B substrates),^27, 28^ and PTP1B we observed a loss of signal when cells were treated with the inhibitor (Figure 4b, left). Each dot represents colocalization between PTP1B and Src and PTP1B and EGFR, respectively. The ImageJ (NIH, USA) program was used to quantify the number of dots in non-treated *versus* treated cells (Figure 4b, right).

### PTP1B inhibitor induces loss of adhesion molecules in D492

**Figure 5:**
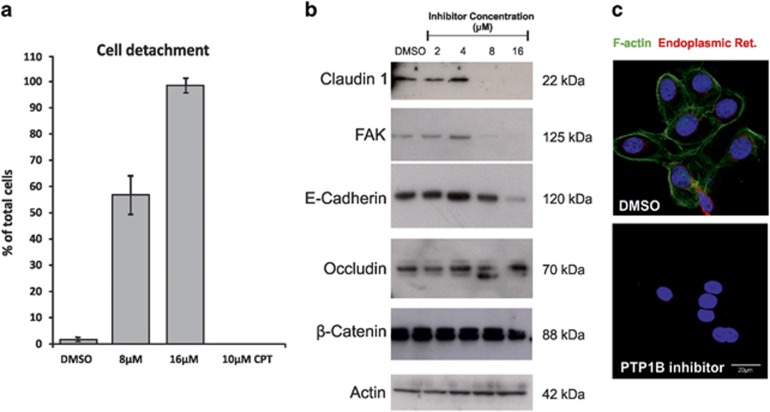
PTP1B inhibition affects expression of cell adhesion molecules and actin polymerization. (**a**) D492 cells treated with PTP1B inhibitor show loose surface attachment. D492 cells were treated with 8 or 16 *μ*M of the PTP1B inhibitor, 10 *μ*M of CPT or DMSO as control for 24 h, and assayed for sensitivity to trypsinization. (**b**) Expression of various cell adhesion molecules is reduced in D492 cells after PTP1B inhibition for 48 h. D492 cells were treated with various concentrations of the PTP1B inhibitor for 48 h followed by protein isolation. Claudin-1, FAK and E-cadherin expression is lost when treated with 16 *μ*M of the inhibitor. PTP1B inhibition does not affect occludin, *β*-catenin and actin expression. (**c**) F-actin polymerization is lost in D492 cells treated with inhibitor for 5 h. Phalloidin stains F-actin (green), Concavalin A stains endoplasmic reticulum (red)

To investigate how PTP1B inhibition affects cell adhesion, we used a cell detachment assay where cells were treated with 8 or 16 *μ*M of the PTP1B inhibitor, 10 *μ*M of CPT or DMSO as control for 24 h (Figure 5a). After treatment, cell attachment to the culture plate was measured as cells detached after 1 min of trypsinization compared with total number of cells. PTP1B-treated cells were much more sensitive to trypsinization than control cells. In contrast, induction of apoptosis with CPT did not facilitate detachment from the culture plate.

To further confirm our hypothesis that the PTP1B inhibitor is affecting cell adhesion, we treated D492 cells with different concentrations of the PTP1B inhibitor and tested the expression of selected adhesion molecules by western blot (Figure 5b). At the highest concentration of the inhibitor, cells lost the expression of claudin-1, FAK and E-cadherin, whereas occludin expression remained unchanged. Moreover, phalloidin staining shows that actin polymerization in D492 cells is completely abolished after treatment with the PTP1B inhibitor (Figure 5c), whereas total actin levels are unaffected (Figure 5b). To supplement this data, we treated HMLE cells with various concentration of the inhibitor for 48 h and tested the expression of E-cadherin, FAK and pFAK^Y925^ (Supplementary Figure 4). Western blot analysis shows diminished expression of E-cadherin in HMLE cells treated with 16 *μ*M of the PTP1B inhibitor, reduced expression of FAK and loss of FAK phosphorylation.

### EMT sensitizes cells to loss of attachment caused by PTP1B inhibition

During EMT, epithelial cells loose cell–cell adhesion and gain increased migratory and invasive properties. To test the effect of the PTP1B inhibitor on less adherent cells than D492, we used D492M, a mesenchymal subline of D492. D492M is ideal to use as a comparison as it is directly derived from D492 and has all the characteristics of a cell line that has gone through EMT.^29^

**Figure 6:**
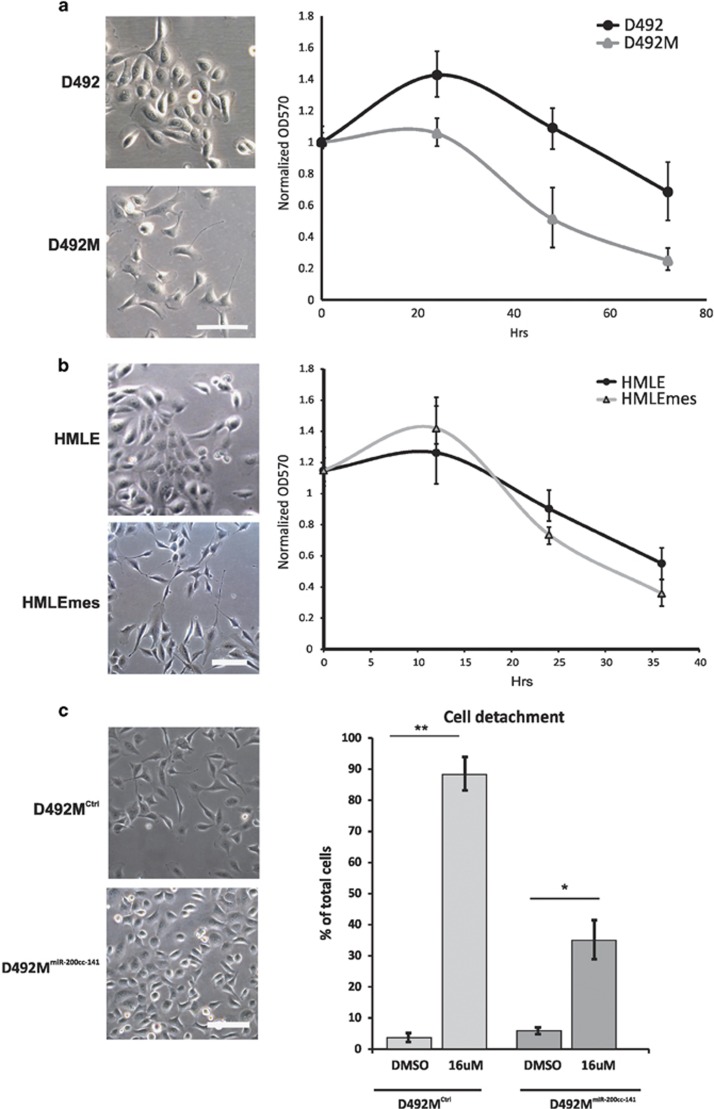
Cells with mesenchymal phenotype are more sensitive to PTP1B inhibition than their isogenic epithelial counterparts. (**a**) D492M is more sensitive toward PTP1B inhibition than D492. The isogenic cell lines D492M and D492 that show mesenchymal and epithelial phenotype, respectively, were treated with DMSO (control) or 16 *μ*M of a specific PTP1B inhibitor for 3 days. Cells were stained with crystal violet at different time points and optical density measured at 570 nm to evaluate the survival of cells. Note how D492M cells show lesser survival than D492. All experiments were conducted in triplicate. (**b**) HMLEmes is more sensitive to PTP1B inhibition than HMLE. HMLE and HMLEmes were treated with DMSO (control) or 16 *μ*M of a specific PTP1B inhibitor for 3 days, stained with crystal violet and the optical density at 570 nm determined. (**c**) MET reverses sensitivity to PTP1B inhibition in D492M. D492M^Ctrl^ (mesenchymal) and D492M^miR-200c-141^ (epithelial) were treated with 16 *μ*M of the PTP1B inhibitor or DMSO as control for 24 h and assayed for sensitivity to trypsinization

D492M has a mesenchymal phenotype, is more mobile and has reduced attachment to the culture flask compared with D492. We compared the effects of the inhibitor on D492 and D492M using a crystal violet survival curve. Importantly, this assay does not assess cell death, but how many cells are attached to the culture flask surface in each measured time point. Figure 6a shows a significantly reduced cell count in D492M compared with D492, and thus, an increased effect of the PTP1B inhibitor. To show that the increased effects of PTP1B inhibition on mesenchymal cells are not cell line specific, we used the human mammary cell line HMLE^30^ and its mesenchymal derivative HMLEmes. The mesenchymal-derived cells were made by sorting for the EpCAM-negative population of the HMLE cell line. HMLEmes show classical characteristics of EMT in gene expression and morphology (unpublished data). As with the D492 cell lines, HMLE and HMLEmes are not cultured in the presence of serum. Similar to D492/D492M, the mesenchymal cell line HMLEmes also proved to be more vulnerable to PTP1B inhibition than the epithelial HMLE cell line, indicating that cells with a mesenchymal phenotype and less expression of cell adhesion molecules are more sensitive to PTP1B inhibition (Figure 6b).

We have previously shown that overexpression of miR-200c-141 in D492M induces mesenchymal to epithelial transition (MET).^31^ To analyze if MET makes cells more resistant to cell death after PTP1B inhibition, we used a cell detachment assay to compare the differences between D492M^Ctrl^ and D492M^miR-200c-141^ with regard to trypsinization. Figure 6c shows that D492M^Ctrl^ is more sensitive to trypsinization after treatment with the PTP1B inhibitor than its epithelial subline, D492M^miR-200c-141^.

Collectively, we have shown that breast epithelial cells with a mesenchymal phenotype are more sensitive to PTP1B inhibition than their epithelial counterparts.

## Discussion

In this work, we have studied the expression of PTP1B in human breast tissue and human breast epithelial progenitor cells. We have furthermore analyzed its role in cell proliferation and survival of breast epithelial cells in culture. PTP1B expression is abundant in primary human breast tissue with highest expression in primary basal/myoepithelial cells and fibroblasts. Expression of PTP1B is also high in D492 cells grown in 3D rBM where staining is increased at lobular ends of the branching structures. Inhibition of PTP1B via a specific PTP1B inhibitor or by siRNA knockdown of PTP1B resulted in marked effects on the survival of D492 cells by inducing cell death. Our data based on CFSE staining further indicates that the cell death in D492 is primarily in dividing cells. Furthermore, there were striking changes in the cell morphology during PTP1B inhibitor treatment; the cells were round with characteristics of reduced cell attachment and spreading. Collectively from these changes, we conclude that inhibition of PTP1B in D492 cells results in loss of cell attachment and cell death in the form of anoikis. In the current work, we have exclusively focused on *in vitro* models. Culturing cells in 3D rBM can capture morphogenesis seen *in vivo* but will never fully replace *in vivo* models. Therefore, it will be important to continue this work using *in vivo* models.

Our results in the breast gland are consistent with other publications where PTP1B inhibition resulted in apoptosis in non-small cell lung cancer cells^22^ and susceptibility to anoikis in colorectal cancer cells.^32^ We show here that the cell death induced by inhibition/knockdown of PTP1B and CPT-induced apoptosis demonstrates morphology representative of anoikis and classical apoptosis, respectively. We also provide evidence that PTP1B can activate Src, a well-known oncogene, which is known to have a role in anoikis.^33, 34^ Furthermore, PTP1B inhibition results in downregulation of the adhesion molecules claudin-1, E-cadherin and FAK and disrupted actin polymerization. Interestingly, mesenchymal derivatives of mammary epithelial cells (both D492M and HMLEmes) are more sensitive to PTP1B inhibition than the epithelial cell lines. Moreover, a MET cell line, D492M^miR-200c-141^ is more resistant to PTP1B inhibition than the control cell line. These data are particularly interesting because cells that have undergone EMT, especially cancer cells, are in general more resistant against drug treatment. If inhibition of PTP1B renders these cells more vulnerable for induction of cell death, this could open up possibilities of using PTP1B inhibitors in therapy against a subset of breast cancer tumors, namely those enriched with cells showing an EMT phenotype.

Anoikis resistance of tumor cells constitutes an essential event in tumor progression to metastases in carcinomas.^35^ The metastatic process involves the detachment of adherent cancer cells, invasion into the surrounding stroma and extravasation of the metastatic cells into vascular vessels. Anchorage-independent cells then travel to distant organs where they can colonize and form metastasis. Studies of anoikis and the characterization of the mechanisms mediating sensitivity to anoikis can provide important insights into epithelial homeostasis and metastasis. Src tyrosine kinase is one of the key molecules believed to have a critical role in the development of resistance against anoikis. Studies have shown that Src overexpression significantly contributes to resistance of epithelial cells to anoikis.^36, 37^ Src is a powerful oncogene but it is rarely mutated in human cancer, suggesting that it is involved in later stages of carcinogenesis and has a supporting, rather than an initiating role.^38^ In human breast cancer, Src activity is often increased 4- to 30-fold compared with activity in normal breast tissue and it has also been reported that Src protein levels can be increased in breast cancer.^39^ Numerous studies have shown that elevated catalytic activity of Src is required to confer resistance to cell death, thus identifying Src as a survival factor that protects cancer cells from apoptosis and anoikis.^40, 41^

Studies indicate that PTP1B is the primary PTP capable of dephosphorylating c-Src in breast cancer cell lines and thus controlling its kinase activity.^42^ Our findings suggest that when D492 cells are treated with the PTP1B inhibitor Src activity is diminished, identifying PTP1B as a positive regulator of Src. Western blotting showed that PTP1B inhibition enhanced phosphorylation on the tyrosine inhibitory phosphorylation site in Src. This finding was further confirmed by staining cells with a Src-phospho-specific (Y529) antibody, where D492 cells treated with PTP1B inhibitor showed increased Y529-phosphorylated Src at the cell membrane, as well as in the cytoplasm. This staining correlates with reported Src localization in other studies.^12^

In this study, we also show that inhibiting PTP1B downregulates adhesion signaling. Thus, PTP1B inhibition could interfere with the cancer cells abilities to reside in a secondary site. Importantly, the mesenchymal D492M cell line is more sensitive to PTP1B inhibition than D492. Mesenchymal cells are characteristically less sensitive to apoptosis and many chemotherapeutic agents.^43, 44^ Mesenchymal cells are furthermore less adhesive. We suggest that under these circumstances of low adhesion, the mesenchymal cells rely on Src activity to prevent anoikis. As PTP1B inhibition leads to reduced Src activity, this may explain the sensitivity of the mesenchymal cells to the inhibitor. These findings suggest a therapeutic role for PTP1B inhibition to target mesenchymal derivatives of cancer cells, cells that are usually less likely to be targeted by conventional chemotherapeutics.

Collectively, our data have relevance regarding PTP1B in normal cell biology and cancer biology. We have shown that PTP1B may have an important role in cell adhesion and anoikis. PTP1B has been studied intensively as a drug target for type-2 diabetes, and already there are several inhibitors in clinical trials. Our data suggest that these inhibitors could also have a therapeutic role in cancer, specifically targeting cells that rely on Src-mediated anchorage independence.

## Materials and Methods

### Cell culture

D492 and D492M were cultured in the chemically defined medium H14 (ref. 45) unless indicated otherwise. HMLE and HMLEmes cells were grown in DMEM/F-12 medium supplemented with 10 ng/ml EGF, 10 *μ*g/ml insulin and 0.5 *μ*g/ml hydrocortisone. HMLEmes cells were generated by sorting and collecting an EpCAM-negative population from HMLE cells. HMLE cells were used with the permission from Dr. Robert Weinberg. MCF10a were cultured in H14 medium. All cell lines were routinely authenticated with genotype profiling.

Primary endothelial cells were isolated as previously described^46^and cultured in EBM basal medium (Lonza, Walkersville, MD, USA), containing 50 IU/ml penicillin and 50 *μ*g/ml streptomycin. The EBM medium was supplemented with 5% FBS. Primary luminal epithelial cells were cultured in the chemically defined medium CDM3.^47^ Primary myoepithelial cells were cultured in the chemically defined medium CDM4.^48^ For 3D cultures, 10 000 D492 cells were plated separately inside reconstituted basement membrane matrix, rBM (Matrigel, Becton Dickinson, Franklin Lakes, NJ, USA). Experiments were carried out in 24-well dishes (Falcon, Corning brand, NY, USA) using 300 *μ*l Matrigel, in which single cells were suspended.

For staining of 3D structures, the Matrigel was dissolved with Matrigel lysis buffer on ice at mild rotation for 20–60 min. Branching structures were then spun down in a pre-cooled rotor at 1000 r.p.m. for 5 min at 2 °C. The structures were then resuspended in 30 *μ*l PBS and left to dry on glass slides.^49^

### Materials

PTP1B inhibitor^50^ was purchased from Calbiochem; EMD Millipore brand, Billerica, MA, USA (#539741) and CPT from Sigma (St. Louis, MO, USA) (#C9911). PTP1B rabbit polyclonal antibody was purchased from R&D Systems (Minneapolis, MN, USA) and PTP1B mouse IgG1 antibody was purchased from BD Bioscience (Fisher Scientific brand, Waltham, MA, USA), Src (phospho Y529), actin, GAPDH and K14 antibodies were purchased from Abcam (Cambridge, UK). K18 and vimentin were purchased from DAKO (Agilent brand, Santa Clara, CA, USA) and *β*4-integrin was from Sigma-Aldrich (St. Louis, MO, USA). F-actin was stained with conjugated phalloidin (Invitrogen, Carlsbad, CA, USA). Isotype-specific secondary antibodies were from Invitrogen as well as TOPRO-3 nuclear counterstain. Specimens were visualized on a Zeiss LSM 5 Pascal laser-scanning microscope (Carl Zeiss, Germany) and Olympus FluoView 1200 (Japan).

### qRT-PCR

Total RNA was extracted with Tri-Reagent (Ambion, Thermo Scientific brand, Waltham, MA, USA). The RNA was DNAase treated and reverse transcribed with hexanucleotide primers using Supercript II and Superscript IV (ThermoFisher, Waltham, MA, USA). Resulting cDNA was used as template for qPCR. Primer pairs and probes from Applied Biosystems (Foster City, CA, USA) (TaqMan) were used for PTPN1 (HS00182260_m1) and GAPDH (4326317E) as an endogenous reference gene.

### PTPN1 knockdown

D492 cells were transfected with Silencer Select pre-designed siRNAs (Ambion, silencer select negative control no. 1 siRNA, silencer select validated siRNA PTPN1 s11506, silencer select validated siRNA PTPN1 s11507 and silencer select validated siRNA PTPN1 s11508). Transfections were done according to the manufacturer´s protocol using Lipofectamine RNAiMAX (Invitrogen, cat. 13778030) in 24-well plates (Corning, New York, USA, cat. 353047). RNA was extracted using Tri-Reagent solution (Ambion, cat. AM9738) according to the manufacturer’s protocol. RNA was reverse transcribed using SuperScript IV Reverse Transcriptase (Invitrogen, cat. 18090050) and expression of PTP1B was assayed on Applied Biosystems 7500 Real-Time PCR System using TaqMan Gene Expression Assays Hs00942477 for PTPN1 and Hs99999905 for GAPDH as a control. Cell proliferation was assayed using IncuCyte Zoom Live Cell Analysis System, Essen Bioscience, Ann Arbor, MI, USA.

### Western blot analysis

Protein lysates were acquired using RIPA lysis buffer supplemented with both phosphatase and protease inhibitor cocktails (Life Technologies, Thermo Fisher Scientific brand, Waltham, MA, USA). Equal amounts (5 *μ*g) of proteins in RIPA buffer were separated on 10% NuPage Bis-Tris gels (Invitrogen) and transferred to a PVDF membrane (Invitrogen). Antibodies used are as follows: E-Cad (BD), PTP1B (BD, Fisher Scientific brand, Waltham, MA, USA), pSrc529 (Abcam), claudin-1 (Zymed, San Francisco, CA, USA), occludin (Zymed), *β*-catenin (Biolegend, San Diego, CA, USA), FAK (Cell Signaling, Danvers, MA, USA), pFAK (Cell Signaling) GAPDH (Abcam) and actin (Abcam) were used. In Figures 1,4 and 5, secondary antibodies were horsesadish peroxidase-conjugated anti-mouse or rabbit (BD Scientific; Fisher Scientific brand, Waltham, MA, USA) used at dilution 1:10 000. The protein bands were visualized using enhanced chemiluminescence (ECL) system (Thermo Scientific, Waltham, MA, USA). In Supplementary Figure 4, secondary antibodies were mouse or rabbit IRDye (Li-Cor, Lincoln, NE, USA) used at 1:10 000 and protein bands were detected using the Odyssey Infrared Imaging System (Li-Cor) and then fluorescent images were converted to gray scale.

All western blots are representative of three separate experiments.

### Cell growth curve with PTP1B inhibitor

D492 cells were seeded at 70% confluency per well in a 24-well plate in growth media. One day after seeding, the medium was replaced with H14 medium and treated with either DMSO or the indicated concentration of inhibitor. For each time point, cells were fixed with 250 *μ*l of crystal violet solution (0.25% crystal violet in 20% methanol). Fixed cells were then incubated in 500 *μ*M lysis solution (33% acetic acid) for 10 min. To quantitate the amount of crystal violet in each sample, the optical density at 570 nm was determined using a spectrophotometer.

### Apoptosis assay

Analysis of annexin V/PI binding was determined using an Annexin V-FITC apoptosis Detection Kit I (BD Pharmingen, Becton Dickinson brand, Franklin Lakes, NJ, USA). The D492 cell line was treated with DMSO or indicated concentrations of PTP1B inhibitor for 48 h. Cells were harvested and resuspended in binding buffer at a concentration 1 × 10^6^ cells/ml. Later, 2.5 *μ*l of Annexin V were added to 100 *μ*l of suspension and incubated at RT in the dark for 15 min. In all, 400 *μ*l of 1 × binding buffer were added to each tube and 2.5 *μ*l of PI just before 10 000 cells from each tube were analyzed in a FACSCalibur, BD Bioscience. Data were processed using FCSexpress (*De novo* software, Los Angeles, CA, USA).

### CFSE-based cell proliferation

Proliferation was assessed with a CellTrace CFSE Proliferation Kit – for flow cytometry (Invitrogen). D492 cells were resuspended in 1 ml PBS with 0.1% BSA at 1 × 10^6^ cells/ml and CFSE dye added to the cells in a final concentration of 0.5 *μ*M. The cells were incubated in a water bath at 37 °C for 10 min, 10 ml of FBS was added to stop the reaction and the cells resuspended in 1 ml of normal growth media and seeded in a 24-well plate. PTP1B inhibitor at a final concentration of 8 *μ*M or equivalent amount of DMSO was added to the cells. The dilution of CFSE was measured by counting 10 000 viable cells with FACSCalibur (BD Bioscience) for 4 consecutive days.

### *In situ* PLA

Colocalization analysis of PTP1B interactions with substrate proteins was studied using a PLA using the Duolink(R) kit (Olink Bioscience, Sigma, St. Louis, MO, USA). D492 cells were treated with 32 *μ*M of the PTP1B inhibitor for 5 h, fixed with 4% formaldehyde, blocked and incubated with primary antibodies in two different combinations: PTP1B mouse (BD, #610139) with Src rabbit (Cell Signaling, #2109) and PTP1B rabbit (R&D Systems, AF1366) with EGFR mouse (BD, #555996) overnight, followed by a standard PLA protocol according to the manufacturer's instruction. Nuclei were counterstained with DAPI and specimens were visualized on a Olympus FluoView 1200. For quantification of PLA signal, ImageJ was used to quantify the number and area of the Duolink puncta, and for all experiments quantifications were performed from at least three images.

### Live cell imaging

D492 cells were seeded directly onto eight-well chamber slides, or embedded in Matrigel and seeded into eight-well chamber slides (BD) at densities of 3000 cells per well in 100 *μ*l Matrigel. After 10 days in culture in a standard incubator, the chamber slide was removed from the incubator, treated with the relevant inhibitor and transferred to an automated inverted microscope (Leica DMI 6000B, Germany) equipped with an environmental chamber, temperature control module and CO_2_ regulator (Pecon, Germany). Images were taken through a 20x objective with a CCD camera (Exi Blue, QImaging, Belmont, CA, USA) at 10 min intervals for 50 h. All microscope and camera controls were programmed through the *μ*Manager software package (UCSF, San Francisco, CA, USA).^51^ Annotated time-laps videos were created with the Fiji software package (NIH) for ImageJ.^52^

### Cell detachment assay

Cells were seeded in 24-well plates, grown to 90% confluency and treated with 8 or 16 *μ*M of the PTP1B inhibitor, 10 *μ*M of CPT or DMSO as control in H14 media. After 24-h incubation, the medium was removed and the cells were washed once with 1 × PBS and incubated with 0.25% trypsin at 37 °C for 1 min. In order to detach the cells from the culture plates, 1 × PBS with 10% FBS was added into the wells to inactivate the trypsin and the detached cells were collected into tubes. The remaining cells were incubated with 0.25% trypsin for 20 min to detach all the cells and collected into fresh tubes. The cells were counted and the data were presented as a percentage of cells detached after 1 min of trypsin incubation to total cells.

### Statistical analysis

All growth curves were performed in triplicate for statistical accuracy. Graphs were created in Excel. Error bars represent the standard deviation of the sample (S.D.). *P*-values below 0.05 were considered significant (**P⩽0.01, *P⩽0.05).
