# Supplementary figures and images for: Inhibition of PTP1B disrupts cell–cell adhesion and induces anoikis in breast epithelial cells
Source: Cell Death Dis. 2017 May 11;8(5):e2769–. doi: 10.1038/cddis.2017.177 (PMC5520702; doi:10.1038/cddis.2017.177)

01:20 hr:min

100  $\mu$ m

DMSO

CPT

PTP1B inhibitor

00:00

00:20

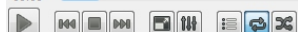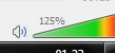

Supplement: Supplementary Figure 1 [file cddis2017177x1.pdf]

01:40 hr:min

100  $\mu$ m

DMSO

CPT

PTP1B inhibitor

00:00

00:14

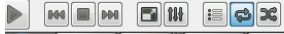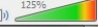

Supplement: Supplementary Figure 2 [file cddis2017177x2.pdf]

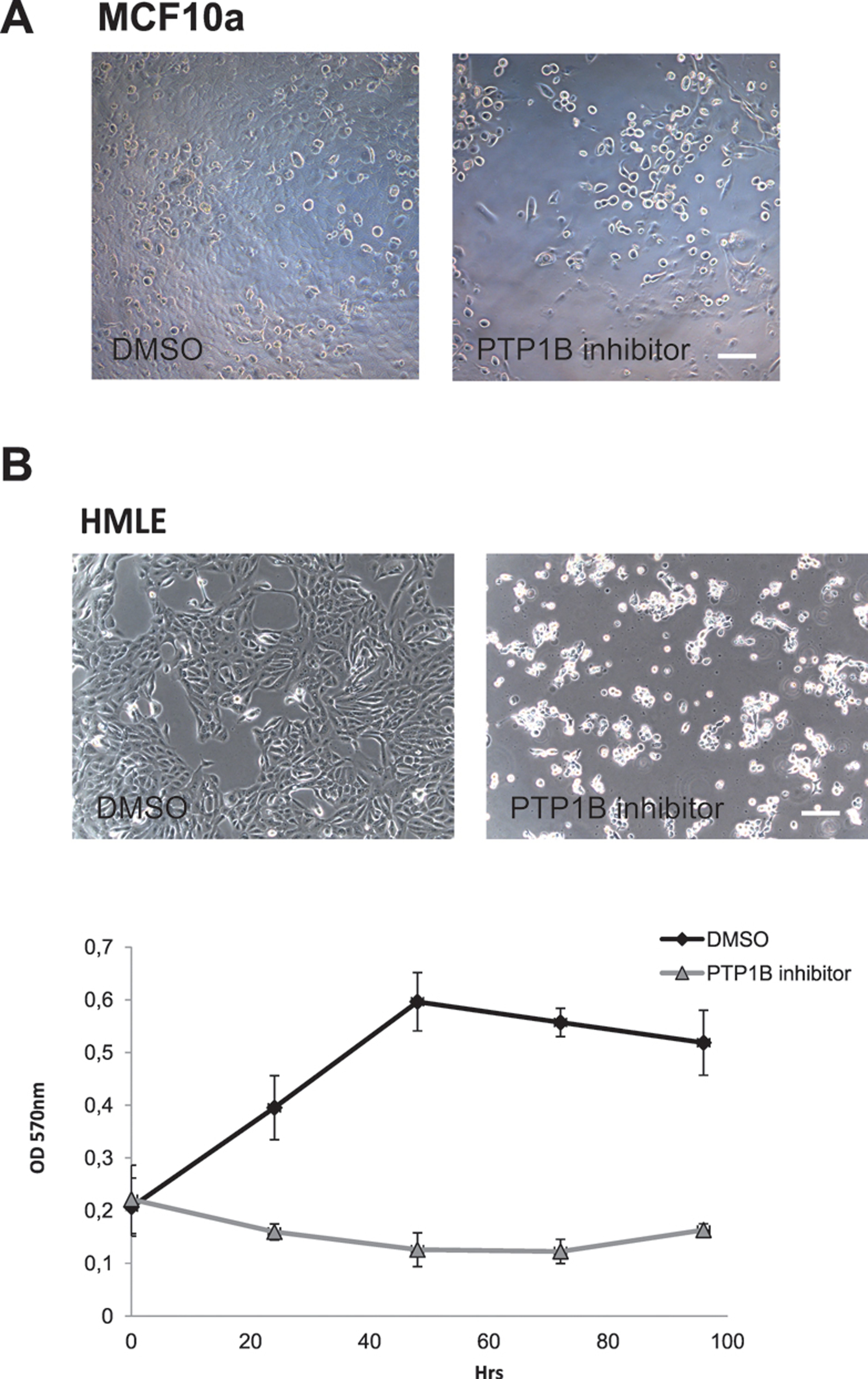

Supplement: Supplementary Figure 3 [file cddis2017177x3.tif]

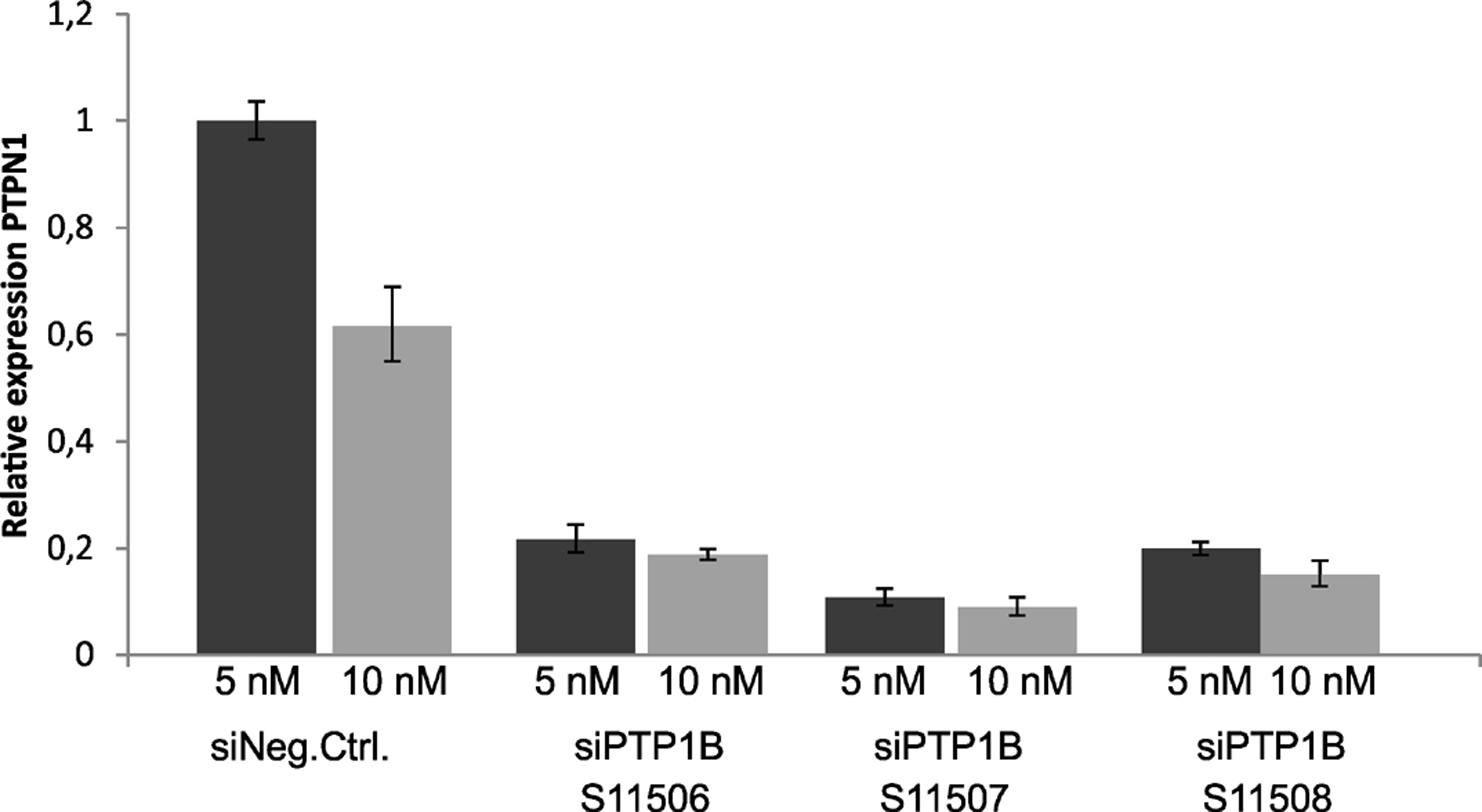

Supplement: Supplementary Figure 4 [file cddis2017177x4.tif]

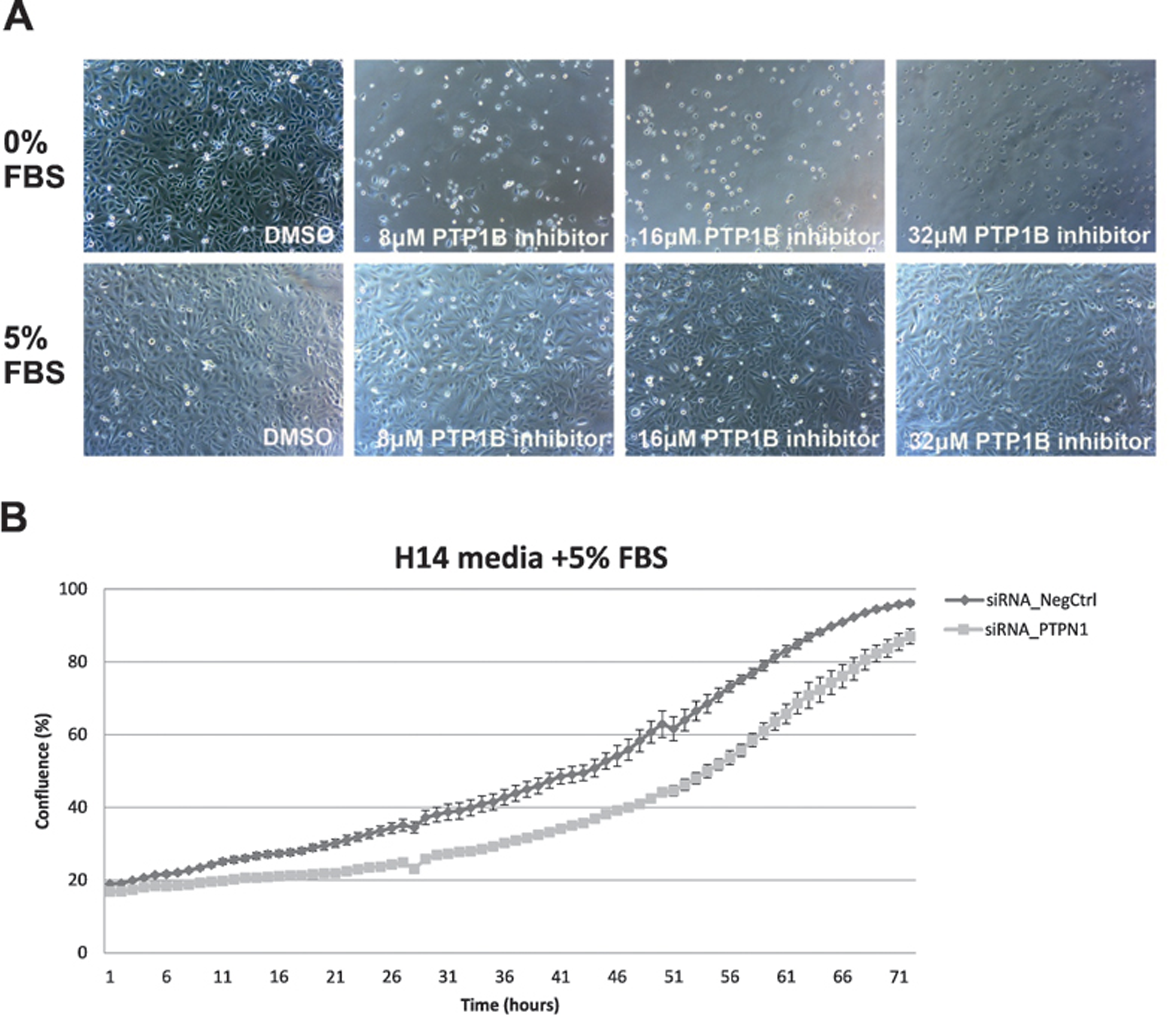

Supplement: Supplementary Figure 5 [file cddis2017177x5.tif]

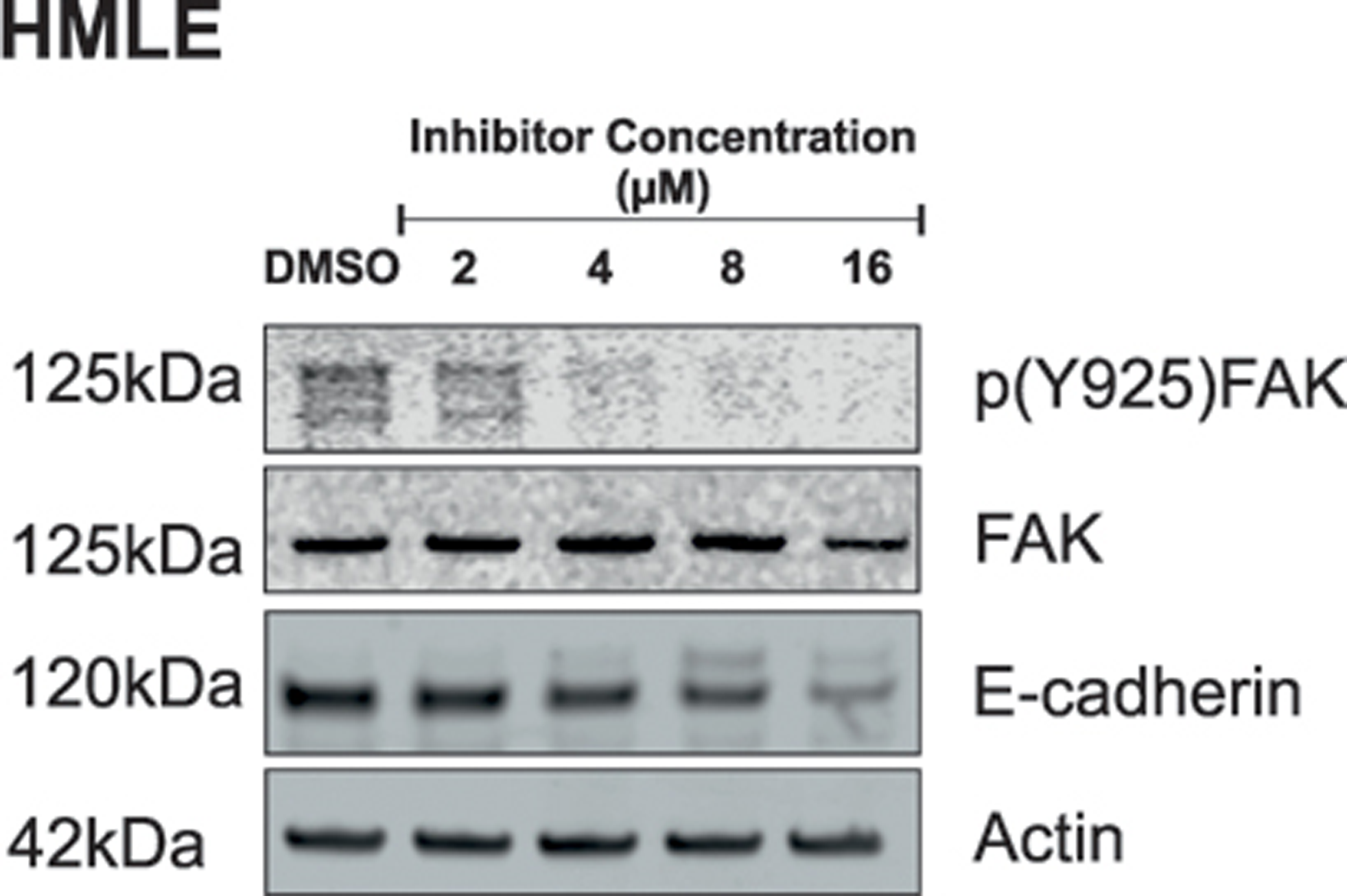

Supplement: Supplementary Figure 6 [file cddis2017177x6.tif]
